# Higher Educational Attainment Associated with Optimal Antenatal Care Visits among Childbearing Women in Zambia

**DOI:** 10.3389/fpubh.2016.00127

**Published:** 2016-06-16

**Authors:** Brian Muyunda, Mpundu Makasa, Choolwe Jacobs, Patrick Musonda, Charles Michelo

**Affiliations:** ^1^Ministry of Health, University Teaching Hospital, Lusaka, Zambia; ^2^Department of Public Health, School of Medicine, University of Zambia, Lusaka, Zambia

**Keywords:** antenatal care, education, visits, Zambia, utilization, pregnant women

## Abstract

**Objective:**

Attendance of at least four antenatal care (ANC) visits over the period of pregnancy has been accepted by World Health Organization to comprise the optimal and adequate standard of ANC because of its positive association with good maternal and neonatal outcomes during the prenatal period. Despite free ANC being provided, many pregnant women have been found not to meet this minimum number of ANC visits in Zambia. We investigated if educational attainment is associated with optimal ANC visits among childbearing women in Zambia.

**Methods:**

Data stem from the 2007 Zambia Demographic and Health Survey for women, aged 15–49 years, who reported ever having been pregnant in the 5 years preceding the survey. The linked data comprised sociodemographic and other obstetrical data, which were cleaned, recoded, and analyzed using STATA version 12 (Stata Corporation, College Station, TX, USA). Multivariate logistic regression was used to examine the association of educational attainment and other background variables.

**Results:**

Women who had higher education level were more likely to attend at least four ANC visits compared to those with no education (AOR 2.8, 95% CI 1.51–5.15; *p* = 0.001); this was especially true in the urban areas. In addition, women with partners with higher education level were also more likely to have optimal ANC attendance (OR 2.0, 95% CI 1.3–3.1; *p* = 0.002).

**Conclusion:**

Educational attainment-associated differentials found to be linked with optimal ANC attendance in this population suggests that access to health care is still driven by inequity-related dynamics and imbalances. Given that inequity stresses are heaviest in the uneducated and probably rural and poor groups, interventions should aim to reach this group.

**Significance:**

The study results will help program managers to increase access to ANC services and direct interventional efforts towards the affected subpopulations, such as the young, uneducated, and rural women. Furthermore, results will help promote maternal health education and advise policy makers and program implementers.

## Introduction

According to World Health Organization (WHO) report, every year in sub-Saharan Africa, more than one million babies die in the first month of life. In 2012, a mortality rate of 48 per 1000 children less than 5 years of age was recorded worldwide. At the same time, 26 million stillbirths were recorded worldwide, of which 98% occurred in low and middle income countries, especially in sub-Saharan Africa (1–3). Many countries in Africa are striving hard to reduce child mortality in an effort to reach the Millennium Development Goal (MDG) number 4, which is to reduce under-five mortality by two-thirds between 1990 and 2015. In addition, it is also estimated that each year in Africa, 30 million women become pregnant, and 18 million give birth at home without skilled care. In 2012 alone, 300,000 women died, and another 289,000 died the following year due to pregnancy-related causes. In the same year, 6.6 million deaths in children less than 5 years of age were recorded, of which 2.9 million deaths occurred during the first 28 days of life as neonates (1, 3, 4). There is more evidence showing increasing risk for preventable challenges. WHO has highlighted this challenge by reporting that tetanus, a preventable illness in pregnancy, kills an estimated 70,000 newborns in Africa every year. This is about 6% of all neonatal deaths each year. Additionally, in Africa, at least 25 million pregnancies are threatened by malaria each year, which results in an estimated 2–15% of maternal anemia. These infections of the placenta result in anemia in the mother and contribute to low birth weight (LBW) and preterm births, which lead to higher infant morbidity and mortality and impaired development of the child. Furthermore, it is estimated that at least 50% of women with acute syphilis suffer adverse pregnancy outcomes (1, 5–9).

Globally, progress has been made in terms of increasing access and use of antenatal care (ANC) as a means for improved pregnancy outcomes. ANC consists of a routine number of scheduled visits aiming at screening and detection of conditions likely to increase adverse effects during pregnancy to ensure healthy outcomes for women and newborn; provide therapeutic interventions and timely treatment of possible complications in pregnancy; and educate pregnant women about planning for safe birth, emergencies during pregnancy, and how to deal with these (10). Through the focused antenatal care (FANC) approach, WHO recommends four ANC visits in low-risk pregnancies, with the first visit in the first trimester, ideally before 12 weeks, but no later than 16 weeks, and the second, third, and fourth visits at 24–28, 32, and 36 weeks, respectively (11). The proportion of pregnant women who book in the first trimester and obtain the recommended minimum number of four visits is still too low. The first consultation is often made late in pregnancy, whereas maximum benefit requires early initiation of ANC within the first trimester (12–16).

Antenatal care is one important component of maternal health services for a healthy pregnancy. In order to help reduce the burden of maternal and new-born mortality, in 2001, WHO issued guidance on a new model of ANC called goal-oriented or FANC for implementation in developing countries. FANC increases the quality of ANC by ensuring that women receive four visits and all the evidence-based interventions that are critical for the well-being of both the child and the mother during and after pregnancy. One of the interventions is the vaccination of first-time pregnant women with the recommended two doses of tetanus toxoid (TT2+). Management and control of syphilis/STIs in pregnant women through universal antenatal screening and treatment is another intervention under FANC. Other interventions include management of preeclampsia, intermittent preventive treatment for malaria in pregnancy (IPTp), and providing insecticide-treated bed nets (ITNs). Additionally, the prevention of mother-to-child transmission of HIV (PMTCT) as well as birth and emergency preparedness at home demand for care all in an effort to reduce both maternal and neonatal mortality (5–7, 17, 18).

In this new strategy of FANC, WHO recommends four ANC visits in low-risk pregnancies and prescribes the evidence-based content for each visit (1, 5, 10, 17–19). It also constitutes screening for health and socioeconomic conditions likely to increase the possibility of specific adverse pregnancy outcomes, providing therapeutic interventions known to be effective, and educating pregnant women about planning for safe birth, emergencies during pregnancy, and how to deal with them (1, 10, 19). Therefore, pregnant women generally are expected to attend an average of four antenatal visits during pregnancy, and they are supposed to schedule their first prenatal appointment as soon as they think they are pregnant. Prenatal tests can provide valuable information about the baby’s health. Doctors might offer ultrasound, blood tests, or other screening tests to detect fetal abnormalities, which if not corrected may lead to complications later.

However, many of the pregnant women do not attend ANC in the first trimester. This means that many pregnant women fail to book for the recommended four ANC visits and are not benefiting from the services offered during this period. Many of them end up with complications leading to high maternal and infant mortality rates. In an effort to determine the factors contributing to underutilization of ANC services by pregnant women, intrapersonal, institutional, and health systems as well as social demographic factors have been identified to be associated with poor ANC attendance (14, 20–23). In addition, there is also strong evidence suggesting that parity, gravidity, knowledge about ANC, intention to get pregnant, and substance use as well as social services and cultural beliefs are some of the factors likely to be associated with late booking and utilization of prenatal care services (13, 16). From the foregoing, there are many sociocultural factors that may be associated with late ANC booking. One of these factors likely to be critical in health promotional activities is educational attainment. Yet, studies focused on examining if education is associated with poor ANC attendance have remained limited. In this study, we aimed to find out if educational attainment is associated with ANC utilization, given the potential impact educational attainment could have on maternal and child survival.

## Methodology

### The 2007 ZDHS Design

The 2007 Zambia Demographic and Health Survey (ZDHS), a nationally representative survey of 7146 women aged 15–49 years and 6500 men aged 15–59 years. The ZDHS used a two-stage stratified sampling. Clusters were selected with probability proportional to size at first stage, and equal probability systematic sampling was applied at second stage. Details of the ZDHS methodology are recorded in the reports (24, 25).

### ANC Attendance Design

The ANC attendance study was based on data that stem from the 2007 ZDHS Women’s Questionnaire. Women who reported having ever been pregnant and ever attended ANC defined the sampling frame for the study (*n* = 7146). From the 7146 women aged 15–49 years who were captured in the survey, 4099 pregnant women who reported attending ANC in the ZDHS (24) comprised the *de facto* eligible sample. Among the records of the *de facto* eligible sample, the information recorded and extracted included the women’s demographic characteristics, their full birth history, and history of ANC for the most recent birth within a 5-year period preceding the survey, including socioeconomic status and educational attainment.

### Statistical Analysis

Both descriptive and inferential statistics were used to examine if educational attainment was associated with ANC attendance. In the first step, univariate analysis (initially by cross tabulations by Pearson’s chi-squared test) and later multiple logistic regression, incorporating survey weights, were performed to examine if educational attainment is associated with ANC attendance and also to control for any confounding or interaction. A *p* value of <0.05 was taken as significant with 95% confidence intervals. The distribution of age as a continuous variable conformed to normality, as assessed by probability plots. Interactions were looked for using the likelihood ratio test, and when identified, they were only reported if considered important in estimating the influence of the background factors. Model diagnostics were done using the Hosmer–Lemeshow goodness-of-fit. The variables in the multivariate logistic regression model were age (grouped and continuous), residence (urban or rural), education, marital status, wealth index, distance to facility, financial constraints, decision-making, intention of pregnancy, timing of first ANC booking, partner age, and educational level as well as maternal and delivery information.

### Ethics

The ZDHS survey obtained ethical approval from the Tropical Diseases Research Centre (TDRC) in Ndola, Zambia and the US Centers for Disease Control and Prevention (CDC) Atlanta research ethics review board (24). Participation in the survey was based on informed and voluntary consent. The reanalysis of the data reported in this study did not infringe on participants’ privacy and was judged by ourselves to pose minimal to no risk, since these data were already anonymized, approved, and made available for public use. In addition to the above ethical measures, we sought a waiver from Excellency in Research Ethics and Science (ERES) Committee that granted us permission to conduct the study on factors influencing maternal and neonatal mortality based on the 2007 ZDHS (Ref. no. 2014-May-023).

## Results

### Participation and Distribution

Overall, of the eligible women who reported using ANC 5 years prior to the survey thus being eligible for the survey (*n* = 4099), the mean age in years was 28 (±7.1), and median age was 27 (IQR = 10). The majority of the participants, 1813 (46%) were in the age range 25–34 years, followed by 1004 (26%) in the age range 20–24 years. The proportion of teenagers aged 15–19 years was only 9% were (351/4099). Distribution by residence showed that 63% were rural women, and 37% were urban. The majority of the women in the study (83%) were married, while only 9% reported not married, see Table 1. Non-participation (not attending ANC at all) was estimated to be less than 5%, given ANC coverage in Zambia estimated by enumerating women who attend ANC at least once is taken to be more than 95%.

**Table 1:** **Basic characteristics of childbearing women attending antenatal clinics in Zambia (24)**.

Characteristic	Frequency	Percent (%)
**Age (years)**
15–19	351	8.9
20–24	1004	25.6
25–34	1813	46.1
35+	762	19.4
Total	3930	100
**Employment status**
Not working	1963	50.2
Working	1946	49.8
Total	3909	100
**Marital status**
Not married	333	8.7
Married	3181	82.9
Living together	20	0.5
Divorced/widowed	305	7.9
Total	3839	100
**Residence**
Rural	2510	63.8
Urban	1420	36.2
Total	3930	100
**Wealth index**
Poor	1671	42.7
Middle	797	20.4
Rich	1447	37
Total	3916^a^	100

*^a^The total sample size reduced (from *n* = 4099 to 3916), because some variables had missing value and were excluded in the analysis and also non-participation due to skip patterns on some questions*.

### Education Attainment

The proportion of women who attended at least four ANC visits was 60% (1590 out of 2509), contrasting the 40% that did not attend less than four ANC visits. Of the total number of women in the study, the majority 64% were in the rural, while 36% were in the urban settlement of the country, see Table 1.

Educational attainment differed significantly between rural and urban groups, the latter having higher educated participants. The pooled mean urban–rural difference was 3.97 (95% CI 3.85–4.09) school years. Many of the participants (61%) reported primary education as their highest level of education attained, while 12% had no education. This was the case irrespective of whether one attended less than four ANC visits or not.

Interestingly, partner education was among the factors associated with the attendance of the recommended number of ANC by pregnant women. Women with partners with higher education level were two times more likely to attend four visits than those whose partners had no education (OR 2.0, 95% CI 1.3–3.14; *p* < 0.002). This likelihood was more prominent in urban areas, where women attend optimal frequency of ANC.

### Other Determinants

Table 2 shows additional factors that were associated with ANC attendance. For those who attended less than four ANC visits, about 48% had intended to get pregnant, while 31% did not. This was significantly differing with the group who attended at least four visits (56%) having intentions to get pregnant (*p* < 0.0001). Timing of the first ANC visit was also another predictor variable associated with attending recommended ANC visits. For the women who attended less than four visits, only 6% reported having booked their first ANC visit in the first trimester, compared to 26% among those who attended at least four ANC visits. For those who had their first booking in the second trimester, there was no difference in both the groups. About 19% of those who had less than four ANC visits reported having their first booking in the third trimester, compared to 1% of those who had at least four ANC visits. This difference was statistically significant, as shown by an extremely strong association of *p* value (*p* < 0.0001).

**Table 2:** **Description of background factors associated with ANC attendance of at least four visits during pregnancy in Zambia – results of Pearson’s chi-squared test (24)**.

Less than four ANC		At least four ANC	
Characteristic	*n* (%)	*n* (%)	*p* value
**Age**			0.0028
15–19	152 (10.3)	179 (7.3)	
20–24	397 (27.0)	587 (24.1)	
25–34	646 (43.8)	1164 (47.7)	
35+	279 (18.9)	512 (20.9)	
Total	1474 (100.0)	2442 (100.0)	
**Education**			0.0011
No education	199 (13.5)	289 (11.9)	
Primary	915 (62.1)	1479 (60.6)	
Secondary	338 (22.9)	577 (23.6)	
Higher	22 (1.5)	98 (4.0)	
Total	1474 (100.0)	2442 (100.0)	
**Parity**			0.9844
1	284 (19.3)	465 (19.1)	
2 and 3	480 (32.6)	793 (32.5)	
4+	709 (48.2)	1183 (48.5)	
Total	1474 (100.0)	2442 (100.0)	
Intention of pregnancy*			<0.0001
Then	702 (47.6)	1368 (56.0)	
later	454 (30.8)	629 (25.7)	
No more	318 (21.6)	445 (18.2)	
Total	1474 (100.0)	2442 (100.0)	
**Partner education**			0.0029
No education	108 (8.5)	152 (6.8)	
Primary	677 (50.6)	1052 (46.9)	
Secondary	488 (36.5)	847 (37.7)	
Higher	66 (4.9)	194 (8.6)	
Total	1338 (100.0)	2245 (100.0)	
**Partner age**			0.0067
15–24	115 (9.5)	124 (6.2)	
25–34	524 (43.3)	901 (45.2)	
35+	571 (47.2)	967 (48.6)	
Total	1210 (100.0)	1992 (100.0)	
**First ANC visit**			<0.0001
First trimester	83 (5.6)	678 (27.8)	
Second trimester	1111 (75.4)	1737 (71.1)	
Third trimester	280 (19.0)	26 (1.1)	
Total	1474 (100.0)	2442 (100.0)	
Distance*			0.0983
A big problem	709 (48.1)	1076 (44.1)	
Not a problem	765 (51.9)	1366 (55.9)	
Total	1474 (100.0)	2442 (100.0)	

*Intention of pregnancy then meant pregnancy was planned and wanted then. Later meant a pregnancy was planned for a later time in future, and no more meant never wanted anymore children and pregnancy was a mistake. Distance being a big problem meant woman had to cover very long distance from home to access ANC services, and not a big problem meant otherwise.

The women whose partners had attained higher education level were two times more likely to attend four visits than those whose partners had no education (OR 2.0, 95% CI 1.3–3.14; *p* < 0.002). Women in the later stages of their pregnancy tended to be less likely to attend ANC after the initial booking (second trimester, OR 0.19, 95% CI 0.15–0.25; *p* < 0.0001; third trimester, OR 0.01, 95% CI 0.007–0.02; *p* < 0.0001).

Furthermore, we also observed that the higher the age of the woman, the more likely the woman would attend the recommended number of ANC visits. Women in the age-group 35 and above were 1.7 times more likely to attend at least four ANC visits compared to the age-group 15–19 years. This is shown in Table 3.

**Table 3:** **Key predictors of ANC attendance for at least four visits during pregnancy in Zambia – results of multivariate logistic regression (24)**.

Characteristic	OR	95% CI	*p* value	AOR^a^	95% CI	*p* value
**Age**
15–19	1			1		
20–24	1.26	(0.96–1.66)	0.096	1.1	(0.81–1.48)	0.321
25–34	1.54	(1.18–2.00)	**0.001**	1.3	(1.00–1.79)	0.067
35+	1.56	(1.17–2.09)	**0.003**	1.7	(1.14–2.21)	**0.006**
**Education**
No education	1			1		
Primary	1.11	(0.89–1.39)	0.359	1.3	(0.98–1.58)	0.076
Secondary	1.17	(0.90–1.53)	0.234	1.4	(1.04–1.92)	**0.017**
Higher	3.04	(1.68–5.53)	<**0.0001**	2.8	(1.51–5.15)	**0.001**
**First ANC visit**
First trimester	1			1		
Second trimester	0.19	(0.15–0.25)	<**0.0001**	0.2	(0.15–0.26)	<**0.0001**
Third trimester	0.01	(0.007–0.02)	<**0.0001**	0.01	(0.007–0.020)	<**0.0001**
**Intention of pregnancy**
Then	1			1		
Later	0.71	(0.60–0.85)	<**0.0001**	0.8	(0.67–0.98)	**0.027**
No more	0.72	(0.59–0.88)	**0.0001**	0.9	(0.68–1.06)	0.158
**Residence**
Rural	1			1		
Urban	1.1	(0.98–1.32)	0.258	1.3	(1.05–1.52)	**0.015**
**Distance**
A big problem	1					
Not a problem	1.18	(1.02–1.37)	**0.027**			
**Working**
No	1					
Yes	1.18	(1.01–1.38)	**0.037**			
**Marital status**
Not married	1					
Married	0.94	(0.34–2.63)	0.907			
Divorced	1.46	(0.87–2.43)	0.152			
Widowed	1.38	(0.92–2.07)	0.123			
Not living together	1.14	(0.65–1.98)	0.651			
**Parity**
1	1					
2 and 3	1.01	(0.82–1.25)	0.944			
4+	1.02	(0.84–1.24)	0.868			
**Partner age**
15–24	1					
25–34	1.59	(1.21–2.11)	**0.001**			
35+	1.57	(1.16–2.13)	**0.003**			

*^a^The multivariate logistic regression model adjusted for all other variables*.

*Bold text indicates the p values that were <0.05*.

## Discussion

We found evidence suggesting that higher education level of the woman was a very significant and important factor in determining optimal ANC utilization. Furthermore, we observed that differential inequalities, in women with higher education level who attained optimal ANC frequency, were most prominent in urban and older women than rural and young women, respectively. This finding suggests that educated women most likely have adequate knowledge on ANC services and understand the importance of early booking for ANC as well as attending the recommended four visits. Thus, they tend to value ANC services and will take advantage of this compared to women with lower education. However, these educational attainment-associated differentials observed in this population may also suggest that access to health care is still driven by inequity-related dynamics and imbalances with the poor and predominantly rural uneducated groups experiencing limited access. The role of education and access to health services for HIV prevention and malaria control have been reported before in this population (5, 12, 26–29).

Similarly, the significance of the woman’s education in determining optimal ANC attendance has been documented in other regions (9, 14, 16–18, 22, 30–32). However, in Pakistan, one study showed that education did not show any association with the utilization of ANC services (33). Additionally, there is clear evidence that the higher educational level of the partner of the woman has a positive effect on the attendance of ANC in some places. Similarly, in this study, we established the positive effect educational level of the partner has on attendance of ANC (30, 33). This study has also observed that young women, especially teenagers, tend to initiate their first booking late. This is possibly because of lack of knowledge on the importance of early attendance of ANC, attendance of the recommended number of visits as well as experience with pregnancy and childbirth. Similar results have been observed in other studies in the region (15, 22). Studies have also shown evidence that women who have unintended pregnancies tend to underutilize ANC services. We have convincing evidence in this study indicating that women who had no intentions of getting pregnant booked for their first ANC late (12, 34, 35). These findings confirm the assumption that educational attainment has a bearing on the attendance of recommended ANC among pregnant women.

It is possible that selection biases, such as non-participation by not attending ANC as well as absence during the ZDHS survey, could have affected our estimates. However, the magnitude and direction of this effect can only be assessed to some extent like any other similar studies employing such designs. The non-participation, due to refusal to take part in this survey, is almost zero and thus not considered to be any factor at all. Absence was the most significant cause of non-participation, particularly in those that never attended ANC. We think that the ANC coverage could be used as sentinel of magnitude of that selection bias representing those who were absent. However, first, we note that over 95% of women in Zambia attend ANC at least once. This suggests that the number of self-reported pregnant women who do not usually attend ANC is very minimal. It is for this reason that we believe that the effect of this non-participation bias was very minimal and not important in explaining our findings. The challenge women face in Zambia is attending the recommended four or more ANC visits, and this was the focus of this study. Second, we are aware that the nature of the association found in this analysis might be due to uncontrolled confounding, which could result from other different forms of non-participation bias, such as those from misclassification of “missing values.” However, for all the variables where the information was available or easy to compute, we addressed this at analysis by looking for interaction, and when there was any indication of it, controlled for it. However, it is worth noting that although we are confident we have made critical analytical steps as outlined, we may not guarantee that we have adequately dealt with confounding. In addition, this, coupled with the fact that the measurements of some of the key variables included in this analysis were relatively crude, suggests a need for a further and detailed exploration of potential barriers to care. This is so because some of these barriers may not have been collected and measured, given that the data may not have been specifically collected and measured with this study in mind. This is the case in most secondary analysis-based studies. Therefore, there still exist critical limitations in these observed findings arising from the fact that associations may be partly due to unmeasured, inadequately measured, or unknown confounders. Further study with a more critical and wider assessment of the scope of variables included and of the quality of measurement of these variables is still needed. In view of all these considerations, we still argue that although these biases may exist, we are convinced that they may not be important in explaining our findings, as we believe that their size was small, and effect limited and minimal, given the observation that ANC coverage, which is a basis for high power in the study, is very high in this population.

Therefore, we are confident of making correct interpretations, and thus recommend program managers and policy makers in the service, or line ministries concerned with mother and child health, to utilize them and help to better repackage and strengthen access and availability of ANC services especially for rural women. Furthermore, results could also be useful to program implementers in promoting maternal health education in an effort to reach the low-educated women and strengthen the existing involvement of community health workers and safe motherhood action groups to increase awareness among rural women on the benefits of attending optimal ANC, as observed in other studies (29, 36, 37). Furthermore, more qualitative research may be required to explore the associated challenges and seek to understand why some pregnant women do not attend ANC, despite availability of services.

## Conclusion

In this study, we have demonstrated that ANC attendance is still limited and below acceptable levels. In this population, higher educated urban groups were more likely to meet the required levels of ANC attendance. This conversely suggests that the poor rural and low-educated women are missing out on the benefits of adequate ANC attendance, thereby raising questions of inequity in accessing health care in ANC. Thus, there is an urgent need to examine how this problem could be resolved by increasing access to ANC services and direct interventional efforts toward these women through peer strategies in the communities. This, in the end, could help reduce both maternal and neonatal mortality in Zambia.

## Author Contributions

BM participated in the actual conception of the study, drafted the manuscript, cleaned the dataset, and carried out the statistical analysis. CJ participated in the early drafting of the manuscript and interpretation of statistical analysis results. PM participated in the acquisition of data, review of statistical analysis results, and general review of the manuscript. MM participated in the critical review of the manuscript and also in the statistical handling and interpretation. All authors read and approved the final manuscript. CM reviewed the draft manuscript for intellectual content and participated in the interpretation of the research findings.

## Conflict of Interest Statement

The authors declare that the research was conducted in the absence of any commercial or financial relationships that could be construed as a potential conflict of interest.

## References

[1] World Health Organization. Perinatal and Neonatal Mortality for the Year 2000. (2006). Available from: http://whqlibdoc.who.int/publications/2006/9241563206_eng.pdf

[2] The Millennium Development Goals Report 2014. (2014). Available from: http://www.un.org/millenniumgoals/2014%20MDG%20report/MDG%202014%20English%20web.pdf (accessed 08 June 2016).

[3] World Health Organization. World Health Statistics 2014. (2014). Available from: www.who.int/gho/publications/world_health_statistics/2014/en/

[4] World Health Organization. Perinatal Mother Neonatal & Child health (PMNCH) Report 2013. (2013). Available from: www.who.int/pmnch/knowledge/publications/pmnch_report13.pdf

[5] Hamer DH Mwanakasale V Macleod BW Chalwe V Mukwamataba D Champo D Two-dose versus monthly intermittent preventive treatment of malaria with sulfadoxine-pyrimethamine in HIV-seropositive pregnant Zambian women. J Infect Dis (2007) 196(11):1585–94. 10.1086/522142 18008241

[6] Ali AA Adam I Anaemia and Stillbirth in Kassala Hospital, Eastern Sudan. J Trop Pediatr (2011) 57:62–4. 10.1093/tropej/fmq029 20427426

[7] Ali AA Elgessim ME Taha E Adam GK. Factors associated with perinatal mortality in Kassala, Eastern Sudan: a community-based study 2010-2011. J Trop Pediatr (2014) 60:79–82. 10.1093/tropej/fmt075 24052575

[8] Elhassan EM Mirghani OA Adam I High maternal mortality and stillbirth in the Wad Medani Hospital, Central Sudan, 2003–2007. Trop Doct (2009) 39(4):238–9. 10.1258/td.2009.090005 19762581

[9] Adegnika AA Luty AJ Grobusch MP Ramharter M Yazdanbakhsh M Kremsner PG ABO blood group and the risk of placental malaria in sub-Saharan Africa. Malar J (2011) 10:101. 10.1186/1475-2875-10-101 21513504 PMC3098819

[10] Von Both C Fleβa S Makuwani A Mpembeni R Jahn A. How much time do health services spend on antenatal care? Implications for the introduction of the focused antenatal care model in Tanzania. BMC Pregnancy Childbirth (2006) 6:22. 10.1186/1471-2393-6-22 16796749 PMC1557863

[11] Villar J Bergsjo P Carroli G Gulmezoglu M The WHO new antenatal care model; the way forward. Acta Obstet Gynecol Scand (2003) 82(11):1065–6. 10.1034/j.1600-0412.2003.00331.x

[12] Banda I Michelo C Hazemba A Factors associated with late antenatal care attendance in selected rural and urban communities of the copper belt province of Zambia. Med J Zambia (2012) 39(3):29–36.

[13] Cresswell JA Yu G Hatherall B Morris J Jamal F Harden A Predictors of the timing of initiation of antenatal care in an ethnically diverse urban cohort in the UK. BMC Pregnancy Childbirth (2013) 13:103. 10.1186/1471-2393-13-103 23642084 PMC3652742

[14] Adekanle D Isawumi A Late antenatal care booking and its predictors among pregnant women in South Western Nigeria. Online J Health Health Allied Sci (2008) 7(1):1–4.

[15] Magadi MA Madsen NJ Rodrigues RN. Frequency and timing of antenatal care in Kenya: explaining the variations between women of different communities. Soc Sci Med (2000) 51:551–61. 10.1016/S0277-9536(99)00495-5 10868670

[16] Nwosu BO Ugboaja JO Obi-Nwosu AL Nnebue CC Ifeadike CO. Proximate determinants of antenatal care utilization among women in Southeastern Nigeria. Niger J Med (2012) 21:196–204. 23311191

[17] Kayentao K Garner P van Eijk EM Naidoo I Roper C Mulokozi A Intermittent preventive therapy for malaria during pregnancy using 2 vs 3 or more doses of sulfadoxine-pyrimethamine and risk of low birth weight in Africa: systematic review and meta-analysis. JAMA (2013) 309(6):594–604. 10.1001/jama.2012.216231 23403684 PMC4669677

[18] Adam I Elhassan EM Abdel ED Abdel AA Gamal KA A perspective of the epidemiology of malaria and anaemia and their impact on maternal and perinatal outcomes in Sudan. J Infect Dev Ctries (2010) 5(2):83–7. 21389586 10.3855/jidc.1282

[19] Conrad P Schmid G Tientrebeogo J Moses A Kirenga S Neuhann F Compliance with focused antenatal care services: do health workers in rural Burkina Faso, Uganda and Tanzania perform all ANC procedures? Trop Med Int Health (2012) 17(3):300–7. 10.1111/j.1365-3156.2011.02923.x 22151853

[20] Tariku A Melkamu Y Kebede Z Previous utilization of service does not improve timely booking in antenatal care: cross section study on timing of antenatal care booking at public health facilities in Addis Ababa, Ethiop. J Health Dev (2010) 24(3):226–33.

[21] Overbosch G Nsowah-Nuamah N van den Boom G Damnyag L Determinants of antenatal care use in Ghana. J Afr Econ (2004) 13(2):277–301. 10.1093/jae/ejh008

[22] Low P Paterson J Wouldes T Carter S Williams M Percival T. Factors affecting antenatal care attendance by mothers of Pacific infants living in New Zealand. N Z Med J (2005) 118(1216):U1489. 15937524

[23] Ruth E Christine DS Susan S Factors which influence use of prenatal care in low-income racial ethnic women of Los Angeles county. J Community Health (1991) 16(5):289–93. 10.1007/BF01320336 1955579

[24] ZDHS. Zambia Demographic Health Survey 2007 Key Findings. (2007).

[25] Zambia 2010 Census of Population and Housing. (2016). Available from: http://statsghana.gov.gh/docfiles/2010phc/Census2010_Summary_report_of_final_results.pdf (accessed 08 June 2016).

[26] Michelo C Sandoy IF Fylkesnes K Marked HIV prevalence decline in higher educated young people: evidence from population based surveys (1995–2003) in Zambia. J Int AIDS Soc (2006) 20(7):1031–8. 10.1097/01.aids.0000222076.91114.95 16603856

[27] Sichande M Michelo C Halwindi H Miller J Education attainment of head of households associated with insecticide treated net utilization among five to nineteen year old individuals: evidence from the malaria indicator survey 2010 in Zambia. Malar J (2014) 13:378 10.1186/1475-2875-13-378 25245164 PMC4182788

[28] Sandoy IF Kvale G Michelo C Antenatal clinic based HIV prevalence in Zambia: declining trends but sharp local contrasts in young women. Trop Med Int Health (2006) 11(6):917–28. 10.1111/j.1365-3156.2006.01629.x 16772014

[29] Surridge M Kaluba CGD Simfukwe V Filling a Gap in the Referral System: Linking Communities to Quality Maternal Health Care via an Emergency Transport System in Six Districts of Zambia. Report Prepared for Mobilising Access to Maternal Health Services in Zambia (MAMaZ). (2014).

[30] Miles-Doan R Brewster KL The impact of type of employment on women’s use of prenatal-care services and family planning in urban Cebu, the Philippines. Stud Fam Plann (1998) 29:69–78. 10.2307/172182 9561670

[31] Nnasir N White E Factors affecting ANC among reproductive age group urban scatter of karachi, Pakistan. J Pak Med Assoc (2003) 53(2):120–7. 12705483

[32] Matthews Z Mahendra S Kilaru A Ganapathy S Antenatal care, care-seeking and morbidity in rural Karnataka, India: results of a prospective study. Asia Pac Popul J (2001) 16(2):11–28.

[33] Navaneetham K Dharmalingam A. Utilization of maternal health care services in Southern India. Soc Sci Med (2002) 55:1849–69. 10.1016/S0277-9536(01)00313-6 12383469

[34] Erci B. Barriers to utilization of prenatal care services in Turkey. J Nurs Scholarsh (2003) 35:269–73. 10.1111/j.1547-5069.2003.00269.x 14562496

[35] McCaw-Binns A Grenade LAJ Ashley D. Under-users of antenatal care: a comparison of non-attenders and late attenders for antenatal care, with early attenders. Soc Sci Med (1995) 40:1003–12. 10.1016/0277-9536(94)00175-S 7792624

[36] Haines A Sanders D Lehmann U Rowe AK Lawn JE Jan S Achieving child survival goals: potential contribution of community health workers. Lancet (2007) 369(9579):2121–31. 10.1016/S0140-6736(07)60325-0 17586307

[37] Nougtara A Sauerborn R Oepen C Assessment of MCH services offered by professional and community health workers in the district of Solenzo, Burkina Faso: utilization of MCH services. J Trop Pediatr (1989) 35(Suppl 1):2–9. 2754779

